# One year of COVID-19 in dental health services in Norway: psychological impact, risk perceptions and vaccination status

**DOI:** 10.1186/s12913-023-09981-9

**Published:** 2023-09-08

**Authors:** M. Shabestari, V. E. Ansteinsson, E. A. S. Hovden, L. Stangvaltaite-Mouhat, I. Mdala, R. Skudutyte-Rysstad, M. M. Uhlen-Strand

**Affiliations:** Oral Health Centre of Expertise in Eastern Norway (OHCE-E), Oslo, Norway

**Keywords:** Psychological impact, Fear of COVID-19, SARS-CoV-2, Dentistry, Dental public health, Vaccination

## Abstract

**Background:**

Increased psychological pressure on oral healthcare professionals (OHP) due to COVID-19 has been shown, yet little is known about the long-term psychological impacts. We aimed to study the psychological impact of COVID-19 and associated factors including perceived risk and preparedness and vaccination status among OHP in the first year after the lockdown period in Norway.

**Methods:**

A structured questionnaire sent electronically to dentists, dental hygienists and dental assistants inquired experiences and perceptions during the second year following the outbreak in Norway. The questionnaire comprised a COVID-19 fear scale and questions about risk perception, preparedness and vaccination status. Exploratory factor analysis (EFA) and Structural Equation Modeling (SEM) were used to assess psychological impact, perception of risk and preparedness according to vaccination status of the respondents.

**Results:**

The majority of the 708 respondents were female (92.8%), had ten or more years of work experience (67.1%), and worked in public dental clinics (95.9%). Fears and concerns related to COVID-19 were common, 72.6% feared getting infected and 85.4% feared infecting others. Of the 642 respondents who agreed that their workplaces handled the situation well, 55.6% were fully vaccinated. Three factors were retrieved from EFA: Insecurity, Instability and Infection. SEM showed that females were more concerned with Infection, and respondents with long clinical experience were less likely to express fear about Instability. Fully vaccinated individuals felt more insecure about becoming infected, and those agreeing that their workplaces handled the current situation well were concerned with Insecurity.

**Conclusions:**

Despite widespread perception of adequate preparedness and high vaccine coverage, a considerable psychological impact and high levels of fear of COVID-19 were observed among the majority of OHP. Fully vaccinated individuals had a larger psychological burden than not fully vaccinated and those with unknown vaccination status. These findings can inform means and interventions to reduce negative impacts of fear in populations with a high psychological burden.

**Supplementary Information:**

The online version contains supplementary material available at 10.1186/s12913-023-09981-9.

## Introduction

On 12 March 2020, a national lockdown was announced by the Norwegian government, and during the lockdown (13 March – 17 April), routine non-urgent oral care was requested postponed by the dental health services. Healthcare workers (HCW) in both public and private practice are required by the Norwegian law to provide emergency health care to all patients, and the Norwegian Directorate of Health requested the dental public sector to establish an emergency dental service for patients with suspected or confirmed COVID-19. Some of the public clinics were designated temporarily to provide urgent treatment for these patients.

Recent reviews found many reports discussing the risk of infection that oral healthcare professionals (OHP) encounter due to exposure to droplet and aerosols of saliva and blood produced during dental routine procedures. Despite this perception of greater risk, the infection risk of COVID-19 in dentistry remains uncertain due to paucity of data [1]. An international survey reported that rates of symptoms compatible with COVID-19 for OHP were not significantly different to those reported for the general population [2], in line with a study based on seroprevalence of antibodies against SARS-CoV-2 [3], and data from Norwegian health and employment registries support the notion that occupation may be of limited relevance for the risk of severe COVID-19 and hospitalization [4].

In the beginning of the COVID-19 outbreak in Norway, the presumed high risk of viral transmission and a sudden increase in the demand for personal protective equipment (PPE) caused a shortage of PPE in health and dental services. Some dental clinics were consequently required to temporarily close or staff down in the lockdown period, adding to the perceived emergency. However, individuals’ perception of risk is reported to more accurately predict the psychological impact of COVID-19 than objective measures of situational severity [5, 6]. This may explain the substantial psychological impact of COVID-19 among OHP regardless of patient facing [7], and despite the majority perceiving that oral healthcare was managed relatively well during the lockdown period [8]. Furthermore, studies conducted early in the pandemic showed high levels of worries, stress and anxiety related to COVID-19 among HCW [9, 10] and OHP [11–13]. The perceived risk of infection has been reported to contribute to the psychological distress and physical complaints amongst OHP [14–17]. However, little is known about positive and negative individual perceptions and experiences of the pandemic and how this relates to mental health and well-being.

In December 2020, the mRNA SARS-CoV-2 vaccines were granted conditional marketing authorizations by the European Medicines Agency in response to the public health crisis of COVID-19, and vaccination of the adult population in Norway started soon after. A substantial number of studies performed before vaccination have shown an increased psychological pressure on OHP due to COVID-19 [7, 13, 15, 16]. However, information on whether the introduction of vaccines have alleviated the negative psychological impacts among OHP is scarce. In a recent questionnaire-based study among dentists in Turkey, 65% responded that vaccination had not decreased their fear of COVID-19 [18]. Although there may be differences in fear levels between dental hygienists and dental assistants and dentists [19], to the best of our knowledge, there exists little information about the psychological impact of COVID-19 on members of the entire dental team. Therefore, we aimed to study the psychological impact of COVID-19 in 2021, and associated factors including perceived risk and preparedness and vaccination status among OHP in the first year after the lockdown period in Norway.

## Materials and methods

### Study design and participants

The participants in the present study were OHP, i.e., dental specialists, general dental practitioners, dental hygienists and dental assistants who responded to our questionnaire in May 2021, one year after the lockdown period due to the COVID-19 outbreak in Norway. A structured questionnaire was sent electronically via QuestBack to chief dental officers in all counties in Norway, who were asked to distribute the questionnaire among public dental clinics. Invitations to dentists in the private sector were distributed via local units of the Norwegian Dental Association. Three reminders for participation were sent to the relevant distributors, and the data collection ended 31 August 2021. Recruitment was based on voluntary participation among dentists who had received the questionnaire.

### Questionnaire

The self-reported questionnaire was an updated version of a previously published questionnaire [8], based on information provided by Centers for Disease Control and Prevention (CDC), World Health Organization (WHO), Norwegian Institute of Public Health, Ministry of Health in Norway, guidelines provided by the Norwegian counties, and on previous research conducted under SARS epidemic in 2002–2003 [20]. The questionnaire consisted of 4 parts:

I) Background characteristics and vaccine status. This included sex, age, work experience, profession, size of dental clinic, work sector, presence of clinic leader and whether or not the clinic treated patients suspected or confirmed to have COVID-19.
II) Psychological impact was measured by the fear scale originally developed by Ho et al. [20]. The scale was adapted to the COVID-19 outbreak in Norway and its following lockdown by Stangvaltaite-Mouhat et al. (2020) [8] and Uhlen et al. (2021) [7]. It consisted of 18 items including fear of becoming infected, fear of infecting others, fear of family members becoming infected, fear related to working with COVID-19 patients, and fear of death. OHP was asked to respond to each of the 18 items on a 4-point Likert scale (0 completely false, 1 somewhat false, 2 somewhat true, 3 completely true) to assess the 18-items. For statistical analyses the responses were dichotomized into false (points 0–1) and true (points 2–3). For instance, if the respondent chose the answer alternative “somewhat false” to the statement “COVID-19 makes me think of death”, we treated the response as if COVID-19 did not make the recipient think of death. To follow up our previous survey, we compared responses to the fear scale between 2021 and 2020. Data from 2020 was collected using the same questionnaire as published previously [7] except for questions regarding vaccination status which were included in 2021.
III) OHP perception of risk and preparedness was measured using the following four statements: *Dentists/assistants/hygienists have high risk of infection with COVID-19. My workplace has currently adequate infection control equipment. My workplace handles the current situation well. My workplace is well equipped to handle an escalation*. Responses were dichotomized into agree/completely agree versus undecided/disagree/completely disagree after being scored on a 5-point Likert scale as follows: 1 represented the answer completely agree, 2 somewhat agree, 3 undecided, 4 somewhat disagree, 5 completely disagree. Dichotomized responses were then stratified according to vaccinations status and subjected to statistical analysis.
IV) Dental health service management, including treatment of patients suspected or confirmed to have COVID-19.

### Statistical methods

Descriptive statistics in the form of frequency and percentage distributions was used to describe the background characteristics of the respondents. We applied the Pearson chi-square test to assess the association between the background characteristics of the respondents and their vaccination status. Explanatory factor analysis (EFA) was carried out on the 18 items of the COVID-19 fear scale to identify latent constructs using the oblique rotation. The Kaiser Meyer-Olkin (KMO) and the Bartlett’s test of sphericity were used to determine the adequacy of the data for EFA. We obtained a KMO statistic equal to 0.922, which meant that the sample size of 708 respondents was adequate for EFA. Internal consistency of the 18 items of the COVID-19 questionnaire was determined from the Cronbach’s alpha estimate where α ≥ 0.7 was considered acceptable [21]. Using the Kaiser criterion, we retained factors with eigenvalues greater than 1. Structural equation modelling (SEM) was then used to investigate the association between vaccination status and the latent constructs. SEM results were adjusted for the background characteristics listed in Table 1. We used IBM SPSS Statistics 27 to perform descriptive analyses and EFA whereas Stata SE 17 was used for conducting SEM. In all instances, significance was assigned to p < 0.05.

## Results

In total 708 dentists, dental hygienists and dental assistants who worked with patients during the pandemic were included in the analyses. Age, longer work experience and public sector as workplace were positively associated with vaccination status (Table 1).

**Table 1:** Characteristics of the respondents by vaccination status

	Vaccination status n (%)				*p*-value
	Fully vaccinated (n = 385)	Not fully vaccinated (n = 231)	Unknown status (n = 92)	Total (n = 708)	
**Sex**					0.16
Female	351 (53.4)	220 (33.5)	86 (13.1)	657 (92.8)	
Male	34 (66.7)	11 (21.6)	6 (11.8)	51 (7.2)	
Age (years)*					< 0.01
< 30	43 (48.3)	29 (32.6)	17 (19.1)	89 (12.6)	
30–40	105 (46.3)	79 (34.8)	43 (18.9)	227 (32.1)	
41–50	107 (64.8)	46 (27.9)	12 (7.3)	165 (23.3)	
51–60	77 (52.4)	52 (35.4)	18 (12.2)	147 (20.8)	
> 60	53 (66.6)	25 (31.3)	2 (2.5)	80 (11.3)	
Work experience (years)*					< 0.01
0–9	110 (47.2)	78 (33.5)	45 (19.3)	233 (32.9)	
≥ 10	275 (57.9)	153 (32.2)	47 (9.9)	475 (67.1)	
**Profession**					0.18
Dentist	170 (58.0)	94 (32.1)	29 (9.9)	293 (41.4)	
Dental hygienist	70 (49.6)	46 (32.6)	25 (17.7)	141 (19.9)	
Dental assistant	145 (52.9)	91 (33.2)	38 (13.9)	274 (38.7)	
**Size of dental clinic**					0.54
Small (< 7 employees)	82 (58.2)	44 (31.2)	15 (10.6)	141 (19.9)	
Large (≥ 7 employees)	303 (53.4	187 (33.0)	77 (13.6)	567 (80.1)	
Work sector*					0.02
Public	375 (55.2)	220 (32.4)	84 (12.4)	679 (95.9)	
Private	10 (34.5)	11 (37.9)	8 (27.6)	29 (4.1)	
**Clinic leader**					0.25
Yes	64 (61.5)	30 (28.8)	10 (9.6)	104 (14.7)	
No	321 (53.1)	201 (33.3)	82 (13.0)	604 (85.3)	
Does your clinic treat COVID-19 patients?*					< 0.01
Yes	76 (63.9)	39 (32.8)	4 (3.4)	119 (16.8)	
No	309 (52.5)	192 (32.6)	88 (14.9)	589 (83.2)	

*Significant difference in vaccination status in relation to selected background variables

### Psychological impact

Regardless of vaccination status, 90.7% perceived to be at high risk of infection with COVID-19. Similar to the prevalent perception of high risk of infection, high levels of fear of infection were present among the OHP. Compared to our results from 2020, equally high levels of fear and concern were still present in 2021 (Table 2). Most of the respondents (54.4%) were fully vaccinated (two vaccine doses), while 13.0% of the respondents did not disclose their vaccination status. Most of the respondents, despite being fully vaccinated (49.6%), were still insecure about whether they were infected or not. This proportion was significantly higher than 34.8% of respondents who were not fully vaccinated and 15.6% in the group with unknown vaccination status. The proportion of the respondents who felt that the virus was close to them and could invade their bodies at any time was also significantly higher among the vaccinated group (45.1%) compared to the not-fully vaccinated group (36.3%) and of the unknown-vaccination-status group (18.6%). Excluding respondents with unknown vaccination status, the majority of the respondents felt very insecure, and the proportion of the respondents who felt very insecure was significantly higher in the fully vaccinated group (Table S2 in supplementary tables).

**Table 2:** Comparison of responses to fear items between 2020 and 2021

COVID-19 makes me:	Proportions (%)		*p*-value
	2020 (n = 727)	2021 (n = 708)	
1. Fear that I will be infected	72.2	73.6	0.55
2. Fear that I will infect others	87.2	87.9	0.69
3. Feel insecure about whether I have been infected or not	56.9	58.9	0.44
4. Feel that the virus is very close to me and that it can invade my body at any time*	24.2	30.4	0.01
5. Feel very insecure	30.7	30.8	0.97
6. Feel that life is threatening	9.8	10.7	0.57
7. Feel that I have lost control of my life	8.9	11.0	0.18
8. Think of death/ to die	11.7	9.3	0.14
9. Feel that the virus will get out of control and spread continuously	33.1	34.3	0.63
10. Worry about whether my family will be infected	77.7	79.1	0.52
11. Dream that family or colleagues are infected	18.8	17.4	0.49
12. Fear that I will end up in quarantine or be forced to limit my activities*	43.3	48.6	0.04
13. Worry about increased work pressure*	43.9	51.7	0.01
14. Feeling discriminated against by others*	6.7	11.3	0.01
15. Worry about whether my family or friends will keep me at a distance because of my job responsibilities	21.3	24.6	0.14
16. Worry about having to work with COVID-19 patients	44.3	42.5	0.49
17. Worry about other health problems in myself	27.6	32.3	0.05
18. Worry about other health problems in my family members	61.1	57.9	0.22

*Significant difference. Proportions of OHP who responded completely true and somewhat true to the total number of respondents for each fear item

### Perception of risk and preparedness according to vaccination status

Among the respondents, 16.8% worked in clinics that were designated to treat COVID-19 patients. Working in dental practices designated to treat COVID-19 patients was associated with fully vaccinated status. Perceptions about how well equipped the workplaces were in the event of an escalation, were significantly associated with vaccination status: 57.6% (n = 298) of the respondents who agreed or completely agreed that their workplaces were well equipped to handle an escalation, were fully vaccinated. Most of the respondents agreed or completely agreed that their workplace handled the situation well and that their workplace had adequate infection control equipment, 357 (55.6%) and 342 (53.9%) respectively. These perceptions were not different when comparing the fully vaccinated to those not fully vaccinated (Table 3).

**Table 3:** Perceptions of risk and workplace preparedness according to vaccination status

	Vaccination status n (%)				*p*-value
	Fully vaccinated (n = 385)	Not fully vaccinated (n = 231)	Unknown status (n = 92)	Total (n = 708)	
Dentists/assistants/hygienists have high risk of infection with COVID-19					0.91
Agree/completely agree	357 (55.6)	202 (31.5)	83 (12.9)	642	
Undecided/disagree/completely disagree	28 (42.4)	29 (43.9)	9 (13.6)	66	
My workplace has currently adequate infection control equipment					0.73
Agree/completely agree	342 (53.9)	210 (33.1)	82 (12.9)	634 (89.5)	
Undecided/disagree/completely disagree	43 (58.1)	21 (28.4)	10 (13.5)	74 (10.5)	
My workplace handles the current situation well					0.09
Agree/completely agree	357 (55.6)	202 (31.5)	83 (12.9)	642 (90.7)	
Undecided/disagree/completely disagree	28 (42.4)	29 (43.9)	9 (13.6)	66 (9.3)	
My workplace is well equipped to handle an escalation*					0.01
Agree/completely agree	298 (57.6)	162 (31.3)	57 (11.0)	517 (73.0)	
Undecided/disagree/completely disagree	87 (45.5)	69 (36.1)	35 (18.3)	191 (27.0)	

*Significant difference. Distributions and proportions of perceived risk and workplace preparedness according to vaccination status

### Extraction of latent constructs from EFA

Three factors with eigenvalues greater than 1 were extracted and in total, explained 55% of the variance. Factor loadings that were less than 0.50 were excluded from further analyses.

As shown in Table S3, four items of the COVID-19 fear scale loaded high on **Factor 1 (Instability)**: worrying about other health problems in my family members, worrying about whether my family or friends will keep me at a distance because of my job responsibilities, worrying about other health problems in myself and worrying about increased work pressure.

Four items loaded high on **Factor 2 (Infection)**: fear that I will infect others, fear that I will be infected, feeling insecure about whether I have been infected or not and feeling that the virus is very close to me and that it can invade my body at any time. Three COVID-19 items loaded high on **Factor 3 (Insecurity)**: feeling that life is threatening, feeling that I have lost control of my life and thinking of death. The reliability of the latent constructs of the COVID-19 questionnaire using the Cronbach’s alpha ranged between 0.61 and 0.73 (Table S3 in supplementary tables).

### Relationship between extracted factors and vaccination status

Standardized coefficient estimates obtained from the SEM are presented in Table 4. Psychosocial impact factors were significantly associated with background characteristics and vaccination status of OHP. Being fully vaccinated had no bearing on Instability and Infection. However, fully vaccinated individuals were more likely to be concerned with Insecurity. Respondents with unknown vaccination status were more concerned with Infection. Respondents who agreed/completely agreed that their workplaces handled the current situation well were still concerned with Insecurity. Female respondents were more likely to be concerned about Infection.

Respondents with at least 10 years work experience and respondents who agreed/completely agreed that their workplaces were well equipped to deal with any escalation of the current situation were less likely to be concerned with Instability. Dental assistants, clinic managers, those who worked in large dental practices, those who worked in private dental practices and respondents who agreed/completely agreed that their workplaces were well equipped to deal with any escalation of the current situation were less likely to be concerned with Infection.

**Table 4:** Associations between vaccination status and the latent constructs

Characteristics	Factor 1 Instability β (95% CI)	Factor 2 Infection β (95% CI)	Factor 3 Insecurity β (95% CI)
Vaccination status (ref: Not fully vaccinated			
Fully vaccinated	0.03 (-0.05, 0.11)	-0.06 (-0.14, 0.02)	**0.09 (0.01, 0.17)** ^*^
Unknown status	0.01 (-0.07, 0.09)	**0.09 (0.01, 0.17)** ^*^	0.02 (-0.06, 0.10)
Sex (ref: Male)			
Female	0.07 (-0.01, 0.14)	**0.08 (0.003, 0.15)** ^*^	0.04 (-0.03, 0.12)
Work experience in years (ref: 0–9)			
≥ 10	**-0.12 (-0.20, -0.05)** ^**^	-0.07 (-0.14, 0.004)	-0.05 (-0.13, 0.02)
Profession (ref: Dentist)			
Dental hygienist	0.01 (-0.07, 0.10)	-0.08 (-0.16, 0.04)	0.03 (-0.05, 0.12)
Dental assistant	0.05 (-0.04, 0.14)	**-0.14 (-0.22, -0.05)** ^**^	-0.05 (-0.13, 0.04)
Clinic manager (ref: No)			
Yes	-0.01 (-0.09, 0.06)	**-0.13 (-0.21, -0.06)** ^**^	0.07 (-0.01, 0.14)
Size of dental clinic (ref: < 7)			
≥ 7	0.02 (-0.05, 0.09)	**-0.07 (-0.14, -0.002)** ^*^	-0.03 (-0.10, 0.04)
Work sector (ref: Public)			
Private	0.01 (-0.06, 0.08)	**-0.14 (-0.21, -0.07)** ^**^	-0.06 (-0.14, 0.01)
Adequate control equipment (ref: Other)			
Agree/completely agree	0.05 (-0.03, 0.13)	0.01 (-0.07, 0.09)	0.05 (-0.03, 0.13)
Current situation (ref: Other)			
Agree/completely agree	-0.07 (-0.15, 0.004)	-0.04 (-0.11, 0.03)	**0.19 (0.12, 0.27)** ^**^
Escalation (ref: Other)			
Agree/completely agree	**-0.11 (-0.20, -0.03)** ^**^	**-0.08 (-0.16, -0.002)** ^*^	-0.02 (-0.10, 0.06)

*p < 0.05 and **p < 0.01. Standardized coefficients (β) were obtained from SEM analysis. CI = 95% confidence interval

## Discussion

The COVID-19 pandemic has affected the mental health and well-being of people all around the world [22–24]. Similarly, it has been reported that OHP in many countries have experienced anxiety and fear due to COVID-19 [25]. In 2021, we used the questionnaire from 2020 [7] to reassess the psychological impact of the COVID-19 on OHP one year after lockdown. The results showed similar levels of psychological impact among the respondents as in our previous study [7], although individual-level matching of the respondents was not performed.

The majority of Norwegian OHP were still satisfied with how their workplace had handled the COVID-19 situation a year after the beginning of the outbreak. They believed that their workplace was adequately supplied with personal protective equipment, and in addition well equipped to handle an escalation. Compared to our results from data collected shortly after lockdown in 2020, OHP had similar levels of fear of COVID-19, and a more positive perception of their workplace in 2021. However, we can only speculate about situational changes that could explain the observed improvement in perceptions of preparedness despite stable levels of fear in the time between the two questionnaires. Firstly, the supplies of PPE were temporarily low and unpredictable in the first phase of the outbreak, and this had improved by the time the second questionnaire was distributed. It is also reasonable to believe that time to get used to a situation is a contributing factor.

At the time of this survey, half of the respondents reported to be fully vaccinated against COVID-19. The findings indicate that the psychological burden of the COVID-19 on OHP was higher among individuals who were fully vaccinated (two vaccine doses) at the time, than among not fully vaccinated and those with unknown vaccination status. This seemingly unexpected finding can be explained by the positive correlation between the perceived risk of contracting the disease and the intention to get vaccinated [26–28]. Our findings are in agreement with vaccination not reducing the anxiety levels of the majority of dentists [18], and with greater fear of the virus among vaccinated individuals [29]. This interpretation is further supported by the association of the intention of receiving a vaccine among HCW [30], and the general population [31] who agreed that COVID-19 is a severe disease. Our results reflect the distinction between the infection-related risk versus the perceived risk, and suggest that the perceived threat of COVID-19 among OHP may persist despite vaccination, given that other determinants of risk perception are unchanged.

Consistent with our previous study, we found a considerable psychological impact of the COVID-19 pandemic on OHP in Norway, with female respondents more likely to be concerned about Infection, and clinicians with longer work experience less likely to be concerned about Instability. This is in accordance with fear and anxiety being reported more frequently in females [11, 32], and with female dentists experiencing a significant change in the work-life balance during the COVID-19 pandemic [33]. It is also reported that younger dentists have a higher risk of moderate-to-severe fear and anxiety, which might be partly explained by less experienced dentists having more economic concerns than those with longer experience [11]. However, in Norway, the public sector was shielded from layoffs, and the risk of job loss or dismissals is higher the lower the education, income, hourly wage and social class background of an employee [34]. Therefore, it can be argued that economic concerns related to job loss was not a major driver of the psychological impact among OHP in the public sector in Norway.

Regardless of the vaccination status, the perceived risks of infection in the current study population are rather high, but not unique to OHP. Perceptions of COVID-19 as a severe threat to health and the corresponding high levels of fear of consequences have also been observed among healthcare workers [31] and in the general populations [35–37]. In contrast to these risk perceptions, the overall risk of hospital admissions due to COVID-19 among patient facing and non-patient facing HCW in 2020 was ranging from 0.07 to 0.2% respectively [38, 39], and the severity and mortality was significantly lower in HCW than in the general population [40]. In our surveys in 2020 and 2021, approximately 70% of the respondents feared that they themselves will be infected, although the risk of dentists being infected was 0.012% in 2020 [4]. Furthermore, 85% feared that they would infect others and 75% were concerned that their families would get infected (Table 2). One third of the respondents felt very insecure due to COVID-19, and four out of ten OHP worried both in 2020 and 2021 about having to treat patients infected with the virus. These findings show that perceptions of personal risk were widespread. Further research is needed to corroborate the complexity of determinants of fear associated with COVID-19 beyond infection-related risks.

Our data suggest that the respondents’ perceptions remained unchanged between the two surveys, and it can be discussed whether the high levels of fear observed in this study affected vaccination among OHP. Based on relevant literature, it is suggested that personal risk perception promotes change in behavior [12, 41] and adherence to social distancing, quarantine protocols, and vaccine uptake [28, 42–44]. Consequently, persistent levels of personal risk perceived by the respondents may have contributed to vaccine acceptance in our study population.

Fear and the associated negative emotions entail physical and mental health costs [45, 46]. High levels of fear related to COVID-19 can contribute to discrimination [47, 48], which may be the case for 11.3% of our respondents who felt that they were discriminated against although we did not investigate why those respondents had this experience. Fear of infection may also reduce coverage of immunization services, and contribute to restriction on city-wide movements, shortage of workers, and diversion of resources to address the pandemic [49]. Strategies to reduce fear, such as improving sense of self-efficacy and communicating a balanced view of risks may be effective ways to promote public health during epidemics [50]. Furthermore, more focus on individuals with risk factors for mental burden may be necessary to prevent negative psychological impact.

The generalizability of our results may be limited due to potential selection bias, as individuals with high levels of fear may be responding to the questionnaire more often. Another limitation is that response rate could not be calculated as the number of OHP who received the questionnaire was unknown. However, based on the number of responses and national registry data, we estimated that approximately 6% of the total number of registered dentists and 13% of dental hygienists in Norway responded to the questionnaire [51]. These results cannot be generalized to the private dental service as the respondents from the public sector were overrepresented. Among the strengths of our study are the comparability and consistency of the results even though the individual respondents were not matched between 2020 and 2021. Another strength is that inclusion of dental hygienists and dental assistants together with dentists in the study allows the generalizability of the results to the public dental service in Norway.

## Conclusions

Fear of infection for oneself and one’s family seem to persist despite high vaccine coverage among OHP in the public dental healthcare. Individuals among OHP who were fully vaccinated had a larger psychological burden of the COVID-19 than those who were not fully vaccinated and those with unknown vaccination status. In addition, females and clinicians with shorter work experience were more concerned than males and clinicians with longer experience, respectively. Our findings are relevant for the management of COVID-19 and future outbreaks because the psychological impact of COVID-19 among OHP is both substantial and persistent. Because vaccination alone may not reduce fear and the psychological burden in dental healthcare settings, better strategies are needed to reduce the negative impacts of fear, especially among those with the highest psychological burden.

### Electronic supplementary material

Below is the link to the electronic supplementary material.

Supplementary Material 1

## Data Availability

The dataset used in the current study is available from the corresponding author on reasonable request.

## List of abbreviations

HCW: Healthcare workers
OHP: Oral healthcare professionals
COVID-19: Coronavirus Disease 2019
EFA: Exploratory factor analysis
SEM: Structural equation Modeling
PPE: Personal protective equipment
