# Supplementary material for: One year of COVID-19 in dental health services in Norway: psychological impact, risk perceptions and vaccination status
Source: BMC Health Serv Res. 2023 Sep 8;23:972. doi: 10.1186/s12913-023-09981-9 (PMC10486048; doi:10.1186/s12913-023-09981-9)
Supplement: Supplementary file 1 — Supplementary Material 1 [file 12913_2023_9981_MOESM1_ESM.docx]

# Supplementary tables

### Table S1. Comparison of respondents in 2020 vs. 2021

|  | **2020**  **(n = 1257)** | **2021**  **(n = 768)** | ***p*-value** |
| --- | --- | --- | --- |
| **Sex*** |  |  | 0.01 |
| Female | 1106 (89.4) | 704 (93.0) |  |
| Male | 131 (10.6) | 53 (7.0) |  |
| **Age in years** |  |  | 0.94 |
| < 30 | 158 (12.8) | 95 (12.5) |  |
| 30 – 40 | 368 (29.7) | 236 (31.2) |  |
| 41 – 50 | 312 (25.2) | 179 (23.6) |  |
| 51 - 60 | 254 (20.5) | 157 (20.7) |  |
| > 60 | 145 (11.7) | 90 (11.9) |  |
| **Work experience in years** |  |  | 0.67 |
| 0 - 9 | 389 (31.4) | 245 (32.4) |  |
| ≥ 10 | 848 (68.6) | 512 (67.6) |  |
| **Profession*** |  |  | < 0.01 |
| Dentist | 590 (47.7) | 308 (40.7) |  |
| Dental hygienist | 235 (19.0) | 145 (19.2) |  |
| Dental assistant | 412 (33.3) | 304 (40.2) |  |
| **Size of dental clinic** |  |  | 0.07 |
| Small (< 7 employees) | 293 (23.7) | 153 (20.2) |  |
| Large (≥ 7 employees) | 944 (76.3) | 604 (79.8) |  |
| **Work sector*** |  |  | < 0.01 |
| Public | 1134 (91.7) | 727 (96.0) |  |
| Private | 103 (8.3) | 30 (4.0) |  |
| **Clinic leader** |  |  | - |
| Yes | - | 111 (14.7) |  |
| No | - | 646 (85.3) |  |
| **Does your clinic treat COVID-19 patients?** |  |  | 0.13 |
| Yes | 170 (13.7) | 123 (16.2) |  |
| No | 1067 (86.3) | 634 (83.8) |  |

The figures for 2020 and 2021 represent prevalence of each background characteristic presented as n (%).

### Table S2. Responses to fear items according to vaccination status

|  | **Fully vaccinated**  **(n = 385)** | **Not fully vaccinated**  **(n = 231)** | **Unknown status**  **(n = 92)** | ***p*-value** |
| --- | --- | --- | --- | --- |
| **COVID-19 makes me:** | **Agree/SA (%)** | **Agree/SA (%)** | **Agree/SA (%)** |  |
| 1. Fear that I will be infected | 51.8 | 34.4 | 13.8 | 0.07 |
| 2. Fear that I will infect others | 54.5 | 32.2 | 13.3 | 0.66 |
| 3. Feel insecure about whether I have been infected or not* | 49.6 | 34.8 | 15.6 | < 0.01 |
| 4. Feel that the virus is very close to me and that it can invade my body at any time* | 45.1 | 36.3 | 18.6 | < 0.01 |
| 5. Feel very insecure* | 46.3 | 37.2 | 16.5 | 0.01 |
| 6. Feel that life is threatening | 42.1 | 43.4 | 14.5 | 0.06 |
| 7. Feel that I have lost control of my life | 47.4 | 38.5 | 14.1 | 0.42 |
| 8. Think of death/to die | 45.5 | 42.4 | 12.1 | 0.20 |
| 9. Feel that the virus will get out of control and spread continuously | 51.0 | 32.5 | 16.5 | 0.13 |
| 10. Worry about whether my family will be infected | 54.3 | 32.0 | 13.8 | 0.46 |
| 11. Dream that family or colleagues are infected | 48.8 | 34.1 | 17.1 | 0.24 |
| 12. Fear that I will end up in quarantine or be forced to limit my activities | 52.3 | 34.0 | 13.7 | 0.56 |
| 13. Worry about increased work pressure | 54.1 | 32.5 | 13.4 | 0.95 |
| 14. Feeling discriminated against by others* | 40.0 | 40.0 | 20.0 | 0.02 |
| 15. Worry about whether my family or friends will keep me at a distance because of my job responsibilities | 51.1 | 32.8 | 16.1 | 0.36 |
| 16. Worry about having to work with COVID-19 patients | 50.2 | 35.2 | 14.6 | 0.15 |
| 17. Worry about other health problems in myself | 49.3 | 37.6 | 13.1 | 0.13 |
| 18. Worry about other health problems in my family members | 53.9 | 31.0 | 15.1 | 0.12 |

**Agree/SA:** completely agree/somewhat agree. Proportions of respondents who completely agreed or somewhat agreed to each fear item according to vaccination status.

### Table S3. Summary of factor loadings obtained from explanatory factor analysis of the psychological impact of the COVID-19 questionnaire

| **COVID-19 makes me:** | **Rotated Factor Loadings** | | |
| --- | --- | --- | --- |
|  | **Factor 1**  **Instability** | **Factor 2**  **Infection** | **Factor 3**  **Insecurity** |
| 18.Worry about other health problems in my family members | 0.706 |  |  |
| 15.Worry about whether my family or friends will keep me at a distance because of my job responsibilities | 0.635 |  |  |
| 17.Worry about other health problems in myself | 0.608 |  |  |
| 13.Worry about increased work pressure | 0.536 |  |  |
|  |  |  |  |
| 2.Fear that I will infect others |  | 0.735 |  |
| 1.Fear that I will be infected |  | 0.704 |  |
| 3.Feel insecure about whether I have been infected or not |  | 0.595 |  |
| 4.Feel that the virus is very close to me and that it can invade my body at any time |  | 0.507 |  |
|  |  |  |  |
| 6.Feel that life is threatening |  |  | -0.760 |
| 7.Feel that I have lost control of my life |  |  | -0.623 |
| 8.Think of death/to die |  |  | -0.596 |
|  |  |  |  |
| Eigenvalues | 7.206 | 1.483 | 1.214 |
| % of variance | 40.032 | 8.240 | 6.742 |
| Alpha (α) | 0.61 | 0.656 | 0.733 |
